# Hyperbilirubinemia as a Diagnostic Tool for the Prediction of Appendicular Perforation: A Prospective Study

**DOI:** 10.5005/jp-journals-10018-1141

**Published:** 2016-07-09

**Authors:** Divish Saxena, Mrinal Tandon, Yunus Shah, BS Gedam

**Affiliations:** 1 Department of Surgery, NKP Salve Institute of Medical Sciences, Digdoh Hills, Nagpur, Maharashtra, India

**Keywords:** Acute appendicitis, Hyperbilirubinemia, Perforated appendix.

## Abstract

**Background:**

The certainty of diagnosing acute appendicitis in patients presenting with right iliac fossa pain still remains a mystery though acute appendicitis being the commonest surgical procedure done in emergency. In acute appendicitis, serum bilirubin levels are raised due to hepatocellular damage as a result of direct insult caused by Gram-negative bacterial endotoxemia. The need for the study is to conclude whether the serum bilirubin can be considered as a new laboratory marker to aid in the diagnosis of acute appendicitis and if so, does it have the predictive capacity to warn us about appendicular perforation.

**Materials and methods:**

This is a prospective study carried out at rural tertiary healthcare center and includes 213 patients clinically diagnosed as acute appendicitis.

**Results:**

Out of 213 patients, raised serum bilirubin ≥1.2 mg/dl was present in 195 (91.5%) patients, out of which 194 (99.4%) patients had histopathologically inflamed appendix and this difference was statistically highly significant with p-value < 0.0001. In this study, 32 patients had perforated appendix. Out of those, 30 patients had bilirubin ≥ 4 mg/dl and 2 patients had bilirubin level between 1.2 and < 4 mg/dl. Raised serum bilirubin (≥4 mg/dl) was present in 35 (17.9%) patients, out of which 30 (87.7%) patients had perforated appendix.

**How to cite this article:**

Saxena D, Tandon M, Shah Y, Gedam BS. Hyperbilirubinemia as a Diagnostic Tool for the Prediction of Appendicular Perforation: A Prospective Study. Euroasian J Hepato-Gastroenterol 2015;5(2):87-89.

## INTRODUCTION

The certainty of diagnosing acute appendicitis (AP) in patients presenting with right iliac fossa pain still remains an enigma though acute appendicitis being the commonest surgical procedure done in emergency. A simple appendicitis can progress to perforation, which increases both morbidity and mortality and surgeons, therefore, prefer to operate when the diagnosis is probable rather than waiting till it becomes obvious.^1^ The accuracy of the clinical diagnosis has been reported between 71 and 97% and it depends on the clinical experience of the treating physician.^2^ To avoid the late complications of AP, a negative appendicectomy rate ranging from 20 to 30% has been accepted by the surgeons.^3,4^ Approximately 6% of the population will suffer from acute appendicitis during their lifetime; therefore, much effort has been directed toward early diagnosis and intervention.^5^ The appendix usually perforates around 48 hours after the onset of acute appendicitis and the delay in presentation is mainly responsible for the majority of perforated appendices.^6^ Obstruction of appendicular lumen by fecoliths is the most common cause of acute appendicitis.^7^ The other causes reported for appendicular lumen obstruction are hypertrophy of lymphoid tissue, inspissated barium from previous X-ray studies, tumors, vegetable and fruit seeds, and intestinal parasites.^6^ This luminal obstruction leads to multiplication of Gram-negative bacteria along with continued secretion of mucus leads to intraluminal distension and increased wall pressure. As a result, lymphatic and venous drainage gets impaired leading to mucosal ischemia with progression to gangrene and perforation.^8^ The Gram-negative bacteria secretes endotoxin which is absorbed in portal circulation and is responsible for damaging hepatocytes thereby raising serum biliru-bin levels.^9^ Elevated serum bilirubin level can help in the early diagnosis of acute appendicitis and in predicting its serious complications, most importantly the perforation. The need for the study is to conclude whether the serum bilirubin can be considered as a new laboratory marker to aid in the diagnosis of acute appendicitis and if so, does it have the predictive capacity to warn us about appendicular perforation.

## MATERIALS AND METHODS

Total 213 cases were studied, out of which 153 were male and 60 were female. Our inclusion criteria are all patients coming to hospital with clinical diagnosis of acute appendicitis and patients who undergo appendectomy. Pregnant females, patients on steroids, immunocompromised patients, patients on chemotherapy for malignancy and appendicular lump were excluded from study. Clinical diagnosis of acute appendicitis was based on symptoms of pain in right iliac fossa, migration of pain to RIF, nausea/vomiting, anorexia, fever and signs of peritoneal inflammation like right iliac fossa tenderness, rebound tenderness and guarding. Total serum bilirubin was investigated in all patients of suspected acute appendicitis. Patients with strong suspicion of acute appendicitis were advised appendectomy. After obtaining consent, patient was operated, and the appendectomy specimen was sent for histopathological examination. The histo-pathology report was considered as the final diagnosis.

## RESULTS

Total 213 patients were studied, out of which, 153 (71.8%) were male and 60 (28.2%) were female. Maximum percentage of patients belonged to age group 21 to 30 years (46%), followed by 31 to 40 years (27.2%) age group. The male: female ratio in the present study was 2.5:1. Out of 213 cases, 209 (98.1%) specimens revealed histopathologically inflamed appendix and 4 (1.9%) specimens did not reveal histopathologically inflamed appendix. So, the negative appendicectomy rate in our study was just 1.9%. Out of four negative appendicectomy, two patients were males and two were females. In one female patient, there was twisted right ovarian cyst; in rest of the three patients the diagnosis could not be made.

Raised serum bilirubin (≥1.2 mg/dl) was present in 195(91.5%) patients, out of which 194 (99.4%) patients had histopathologically inflamed appendix and this difference was statistically highly significant with p-value < 0.0001. In this study, 32 patients had perforated appendix. Out of these 32 patients, 30 patients had serum bilirubin more than 4 mg/dl and 2 patients had serum bilirubin level between 1.2 to 4 mg/dl. Raised serum bilirubin (≥4 mg/dl) was present in 35 (17.9%) patients, out of which 30 (87.7%) patients had perforated appendix and this difference was statistically highly significant with p-value < 0.001.

The sensitivity, specificity and diagnostic accuracy of raised serum bilirubin (≥1.2 mg/dl) in acute appendicitis in the present study was found to be 92.82, 75 and 83.09% respectively.

## DISCUSSION

Acute appendicitis is the most common abdominal emergency presenting to a clinician. Despite the advances in the diagnostic field, the diagnosis of acute appendicitis remains an enigma for the surgeon.^7^ To minimize the late complications of acute appendicitis, the most dreadful being appendicular perforation, surgeons all over the world prefer to perform appendicectomy as soon as possible rather than to wait and watch. A negative appendicectomy rate ranging from 20 to 30% has been accepted by the surgeons worldwide.^3,4^ The rise on serum bilirubin levels in acute appendicitis has been attributed to the sepsis caused by Gram-negative bacteria, most commonly by *Escherichia coli^10^* and its endotoxin leading to portal pyemia.^9^ This causes hepatic parenchymal damage and difficulty in secretion and excretion of bile in biliary canaliculi raising total Serum bilirubin levels.^11^ Elevated serum bilirubin level can, therefore, help in the early diagnosis of acute appendicitis and in predicting its perforation as studies suggest that perforation of appendix occurs around 48 hours after onset of acute appendicitis.^7^

In the present study, out of 213 patients, 195 (91.5%) patients, had raised serum bilirubin, i.e. ≥ 1.2 mg/dl and out of 195 patients 194 (99.4%) patients had histopathologically inflamed appendix and one histopathologically not inflamed appendix.

The sensitivity, specificity and diagnostic accuracy of raised serum bilirubin (≥1.2 mg/dl) in acute appendicitis in the present study was 92.82, 75 and 83.09% respectively. The sensitivity of hyperbilirubinemia (≥1.2 mg/dl) for acute appendicitis in the present study is comparable with study done by Khan S in 2008 and Poras Chowdhary et al in 2013.^12,13^

In the present study, the range of hyperbilirubinemia in cases of perforated appendix was 4 to 4.3 mg/dl which is comparable with study done by Sand M et al.^14^ The negative appendicectomy rate in our study is 1.9% which again is well accepted.

## CONCLUSION

It is concluded from our study that in a patient presenting with right iliac fossa pain with a clinical diagnosis of acute appendicitis. Total serum bilirubin levels can be estimated in all cases and a raised serum bilirubin levels (≥1.2 mg/dl) is having more sensitivity in acute appendicitis along with high diagnostic accuracy. More importantly, the sensitivity, specificity and diagnostic accuracy of serum bilirubin levels more than 4 mg/dl in perforated appendix were found to be very high, i.e. 93.7, 97.1 and 96.6% respectively. Therefore, in patients with high serum bilirubin ≥4 mg/dl, are likely to have perforated appendix and early intervention should be done to reduce the mortality rate. The estimation of total serum bilirubin levels should be included in the battery of investigations to support the diagnosis of acute appendicitis and prediction of appendicular perforation along with clinical parameters.
